# Increased expression of HGF and c-met in rat small intestine during recovery from methotrexate-induced mucositis

**DOI:** 10.1054/bjoc.1999.1023

**Published:** 2000-02

**Authors:** C J Xian, R Couper, G S Howarth, L C Read, N C Kallincos

**Affiliations:** Child Health Research Institute, University of Adelaide Department of Paediatrics and Cooperative Research Centre for Tissue Growth and Repair, 72 King William Road, Adelaide, 5006, Australia

**Keywords:** hepatocyte growth factor, HGF, c-met, methotrexate, intestinal mucositis, regeneration

## Abstract

Chemotherapy or radiotherapy often cause mucosal damage in the gut (gut mucositis) in cancer patients. As a step to investigate mechanisms underlying subsequent intestinal repair, we have examined the expression profiles of hepatocyte growth factor (HGF) and its receptor c-met, two molecules previously implicated in tissue repair, in comparison to the histopathological and proliferative changes in a rat model of methotrexate-induced small intestinal mucositis. Histological analysis of the intestinal specimens revealed crypt loss and villus atrophy with damage maximal on day 5 after methotrexate injection, and normalization of mucosal structure commencing on day 6. Crypt cell proliferation was decreased dramatically on day 3, normalized on day 4 and up-regulated on days 5 and 6. HGF and c-met protein/mRNA expression was up-regulated between days 4 and 7, with the mRNA co-localizing to the crypt and lower villus epithelium. Therefore, following methotrexate injection, a decrease in crypt cell proliferation preceded histological damage, and conversely, crypt cell hyperproliferation preceded mucosal regeneration. Up-regulation of HGF and c-met coincided with crypt hyperproliferation and mucosal recovery, suggesting a role for HGF in intestinal repair following acute injury. The crypt epithelial localization of HGF and c-met implies an autocrine or paracrine mechanism of HGF action. © 2000 Cancer Research Campaign


*British Journal of Cancer* (2000) **82**(4), 945–952
© 2000 Cancer Research Campaign
DOI: 10.1054/ bjoc.1999.1023, available online at http://www.idealibrary.com on IDEAL®

### Increased expression of HGF and c-met in rat small intestine during recovery from methotrexate-induced mucositis

**CJ Xian, R Couper, GS Howarth, LC Read and NC Kallincos**

Child Health Research Institute, University of Adelaide Department of Paediatrics, and Cooperative Research Centre for Tissue Growth and Repair, 72 King William Road, Adelaide 5006, Australia

**Summary** Chemotherapy or radiotherapy often cause mucosal damage in the gut (gut mucositis) in cancer patients. As a step to investigate mechanisms underlying subsequent intestinal repair, we have examined the expression profiles of hepatocyte growth factor (HGF) and its receptor c-met, two molecules previously implicated in tissue repair, in comparison to the histopathological and proliferative changes in a rat model of methotrexate-induced small intestinal mucositis. Histological analysis of the intestinal specimens revealed crypt loss and villus atrophy with damage maximal on day 5 after methotrexate injection, and normalization of mucosal structure commencing on day 6. Crypt cell proliferation was decreased dramatically on day 3, normalized on day 4 and up-regulated on days 5 and 6. HGF and c-met protein/mRNA expression was up-regulated between days 4 and 7, with the mRNA co-localizing to the crypt and lower villus epithelium. Therefore, following methotrexate injection, a decrease in crypt cell proliferation preceded histological damage, and conversely, crypt cell hyperproliferation preceded mucosal regeneration. Up-regulation of HGF and c-met coincided with crypt hyperproliferation and mucosal recovery, suggesting a role for HGF in intestinal repair following acute injury. The crypt epithelial localization of HGF and c-met implies an autocrine or paracrine mechanism of HGF action. © 2000 Cancer Research Campaign

**Keywords:** hepatocyte growth factor; HGF; c-met; methotrexate; intestinal mucositis; regeneration

Gut mucosal damage or mucositis, often experienced by cancer patients as a result of chemotherapy or radiotherapy, can be so severe as to be dose-limiting in the treatment regimens and be the major reason for the patient's hospitalization (Costa and Donaldson, 1979). While the mucosal damage is known to be induced initially as a consequence of a loss of cell mitosis in the epithelium (Trier, 1962; Altmann, 1974), leading to flattening of the villi and absorptive dysfunction (Donaldson and Lenon, 1979), the mechanisms for the subsequent mucosal regeneration remain largely unknown. The current study aimed to examine the relationship between the expression of hepatocyte growth factor (HGF) and its receptor c-met, molecules previously implicated in intestinal tissue repair, with the histopathological and proliferative changes of the intestine in a rat model of methotrexate-induced intestinal mucositis.

HGF is a potent morphogen, motogen and mitogen for a diverse variety of epithelial cells (Schmassmann et al, 1997*a*). Previous studies have demonstrated the mRNA and protein expression of HGF and c-met in various tissues, including the developing digestive tissues (Wang et al, 1994; Kermorgant et al, 1997) and the normal adult stomach, small intestine and colon (Di Renzo et al, 1991; Prat et al, 1991; Wolf et al, 1991). HGF accelerated repair of gastric epithelium, protected against ethanol-induced gastric epithelial injury (Takahashi et al, 1996) and stimulated mucosal regeneration and functional recovery after massive small bowel resection (Kato et al, 1998). As a step to investigate potential roles of HGF in enhancing intestinal repair in chemotherapy-induced intestinal mucositis, the expression of HGF and its receptor was examined in association with the morphological and proliferative changes in methotrexate-induced small intestinal damage and repair in the rat. Methotrexate, an anti-metabolite used for neoplastic treatment regimens, inhibits the enzyme dihydrofolate reductase. The reduced availability of intracellular folate impairs DNA synthesis (Jolivet et al, 1983). The methotrexate-induced intestinal mucositis in rats is characterized histologically by crypt loss, villus fusion and atrophy, capillary dilatation, and a mixed inflammatory cellular infiltrate (Taminiau et al, 1980; Howarth et al, 1996), resembling the gut mucositis experienced as a common side-effect by patients undergoing chemotherapy or radiotherapy (Altmann, 1974).

*Received 18 June 1999*
*Revised 27 September 1999*
*Accepted 28 September 1999*

*Correspondence to*: CJ Xian

### MATERIALS AND METHODS

### Methotrexate time course and intestinal tissue collection

Intestinal mucositis was induced in male adult Sprague Dawley rats by subcutaneous injection in the suprascapular region of methotrexate at a dose of 2.5 mg $kg^{-1}$ once daily for up to 3 consecutive days (Howarth et al, 1996). The rats were maintained ad libitum up to day 10 after the first injection. Rats without methotrexate injection were used as normal controls (day 0). On each day after the first injection, groups of rats ($n$ = 8) were injected intraperitoneally (i.p.) with bromodeoxyuridine (BrdU) at 50 mg $kg^{-1}$ and sacrificed 1 h later by carbon dioxide overdose. Two groups of pair-fed control rats ($n$ = 4), which received no methotrexate injection but consumed similar daily amounts of diet to the methotrexate-injected animals, were killed on day 5 and day 8 for comparison with their methotrexate-injected counterparts. Specimens from the proximal jejunum were freshly collected, either snap-frozen and stored at –80°C, or fixed in methacarn fixative for 2 h prior to routine processing for paraffin-embedding.

All these procedures were approved by the Animal Ethics Committee of the Women's and Children's Hospital, Adelaide, Australia.

### Histopathological analysis of intestinal damage and repair

Transverse sections (4 µm in thickness) of paraffin-embedded proximal jejunal specimens were stained with haematoxylin and eosin (H&E) and examined with a light microscope. A semi-quantitative histological assessment of intestinal damage was utilized to obtain an overall score of damage severity. A total score for the sample was derived from the sum of scores for 11 histopathological criteria as we have previously described (Howarth et al, 1996), including villus fusion and stunting (atrophy), disruption of brush border and surface enterocytes, disappearance of goblet cells, reduction in numbers of mitotic figures, crypt loss/architectural disruption, disruption or distortion of crypt cells, crypt abscess formation, infiltration of polymorphonuclear cells and lymphocytes and dilatation of lymphatics and capillaries. For each criterion, a score was given to represent a mild (score 1), moderate (2), severe (3), or no (0) changes.

Quantitative histological analyses were also conducted to measure the changes in crypt depth and villus height in the jejunal specimens over the time course. Microscopic images were acquired and analysed. For each animal, the height and depth of ten villi and crypts respectively were measured at three tissue levels, each separated by 100 µm, and means of the 30 measurements were calculated for each animal. Although histological damage prevented accurate measurements of villus and crypt lengths in some regions, in the jejunal region where these measurements were made at least 50% of mucosal area was sufficiently intact for reliable determination of villus height and crypt depth at the time with maximal damage.

### BrdU labelling and cell counting

BrdU labelling was used to assess cell proliferation in the jejunal tissues. Immunostaining of BrdU was performed on 3-µm paraffin sections as previously described (Fox et al, 1996) with a mouse anti-BrdU IgG as the primary antibody, a rabbit anti-mouse biotinylated IgG as the secondary antibody, and avidin and biotinylated horseradish peroxidase reagents and diaminobenzidine tetrahydrochloride as enzyme/substrate reagents (Dako, Carpinteria, CA, USA). BrdU labelling index was determined by counting the numbers of positive and the total number of crypt epithelial cells. For each animal, 50 well-orientated full crypts were analysed, and the number of BrdU-labelled cells and the total number of crypt epithelial cells on the left-side halves of the crypts were counted. The number of positive cells was expressed as a % of the total number of epithelial cells to provide the labelling index for that crypt, and the mean of the 50 crypts was calculated as the labelling index for that animal.

### Generation of rat c-met and rat HGF-specific probes

Reverse transcriptase polymerase chain reaction (RT-PCR) was carried out using the following nested primers based on the published rat c-met sequence (Liu et al, 1996): forward primer (a): AACAACGTACGGTGTCTCCAG; reverse primer (b): CAGGATAGGAATCCAGGAGGA. Poly-$A^+$ RNA (1 µg) extracted from rat liver was reverse-transcribed using AMV reverse transcriptase (Boehringer Mannheim, Mannheim, Germany) in conjunction with primer (b). Subsequent PCR amplification of the first strand of cDNA was performed using primer (a) under the following thermocycling regime; 1 cycle at 94°C for 5 min then 30 cycles at 94°C for 2 min, 60°C for 30 s, 72°C for 1 min and a final step at 94°C/2 min, 60°C for 30 s, 72°C for 10 min. PCR products were ligated into the pGEM-T vector (Promega, Madison, WI, USA), and positive clones sequenced to confirm their identity in comparison to the published rat c-met sequence.

The resultant rat c-met pGEM clone was linearized with *Sal*I and a 393 bp $^{32}$P-labelled antisense riboprobe generated with T7 polymerase using a Maxiscript transcription kit (Ambion, Austin, TX, USA). Sense-strand riboprobe was generated by SP6 transcription of the construct linearized with *Hae*III. A human 18S rRNA antisense riboprobe (Ambion) was also prepared for loading control.

A rat HGF riboprobe was prepared by subcloning of an *Eco*RI fragment of the rat HGF cDNA (provided by Professor T Nakamura, Osaka, Japan) into pSP73 vector (Bresatec Ltd, Adelaide, Australia) and subsequent linearization with *Hinc*II before transcription with SP6 polymerase. The sense probe was synthesized by transcription from the same linearized cDNA with T7 polymerase.

### Ribonuclease protection assays

Ribonuclease protection assays (RPA) were carried out to measure changes in HGF and c-met gene expression using an Ambion RPA II kit (Ambion). RNA was extracted from proximal jejunal tissues using the RNA/DNA/Protein separation reagent (Progen Industries, Brisbane, Australia). Total RNA (60 µg) was hybridized overnight at 45°C with a molar excess of radiolabelled antisense HGF or c-met riboprobe together with 18 S probe. Hybrids were subsequently digested with RNAase A+T1 for 30 min at 37°C and were run on 6% polyacrylamide gels before gels were exposed for autoradiography for between 4 h (for 18S ribosomal RNA signal) and 3 weeks (for HGF signal) at –80°C. Densitometry of autoradiographs was carried out, and abundance of the target gene was expressed as the ratio of the density value of the protected target band over that of the 18S rRNA loading control of the sample.

### In situ hybridization detection of HGF and c-met mRNA

A non-radioactive in situ hybridization approach was used to localize any changes in HGF and c-met mRNA expression during the intestinal damage and repair. Digoxigenin (DIG)-labelled sense and antisense riboprobes were generated similarly as for the ribonuclease protection assays but using a DIG RNA labelling kit (Boehringer Mannheim). In situ hybridization was performed on 4-µm paraffin sections of the proximal jejunum following a protocol as described (Zhou et al, 1999). Hybridization was carried out for 18 h at 55°C with 0.5 µg $ml^{-1}$ the sense or antisense probe. Hybridized probes were detected with an alkaline phosphatase-coupled sheep anti-DIG IgG (Boehringer Mannheim).

 © 2000 Cancer Research Campaign

### Western blotting of HGF and c-met

To demonstrate potential changes in HGF and c-met expression at the protein level, Western blotting was performed on total protein samples isolated from the proximal jejunal tissues, the same pieces of specimens used for total RNA extraction by the RNA/DNA/Protein separation reagent as described above. Equal amounts of protein (200 μg) from each sample, as quantitated using Bradford Reagent (Sigma), and 2 μg of a broad range of biotinylated protein molecular weight markers (Bio-Rad, Hercules, CA, USA) were treated with a reducing sample buffer (Laemmli, 1970), separated on a sodium dodecyl sulphate polyacrylamide gel electrophoresis (SDS-PAGE) mini-gel (10% for HGF, 7.5% for c-met), and electroblotted onto a 0.2 μm nitrocellulose filter. For HGF gel, 200 ng of recombinant human HGF (provided by Professor T Nakamura, Osaka, Japan) was used as a standard. The filters were probed with a goat anti-human HGF IgG (R&D Systems, Minneapolis, MN, USA), or a rabbit anti-mouse c-met IgG (Santa Cruz Biotechnology, Santa Cruz, CA, USA), and were respectively incubated with a rabbit anti-goat biotinylated IgG or a swine anti-rabbit biotinylated IgG (Dako). After incubation with avidin and biotinylated horseradish peroxidase reagents (Dako), filters were developed for enzyme-chemiluminescence signal using ECL reagents (Amersham, Buckinghamshire, UK).

### Statistical analysis

Results of the villus height and crypt depth measurements (mm), BrdU labelling (%) and RNA protection assays (% of day 0 control) between the groups of animals treated with methotrexate and untreated normal controls were compared by one-way analysis of variance using Fisher's protected LSD test.

### RESULTS

As we have observed in earlier studies (Howarth et al, 1996), methotrexate treatment induced typical intestinal histological damage profiles, with the most pronounced effect in the duodenum and proximal jejunum, and least severe damage in the distal ileum. In the proximal jejunum, methotrexate induced readily apparent damage by day 3 (Figure 1B) and maximal damage by day 5 (Figure 1C). Some improvement was noted by day 6, followed by a rapid recovery on day 7 (Figure 1D and Figure 2A). On day 5, jejunal histology was characterized by crypt loss, villous atrophy, fusion and shortening (Figure 1C). On day 6, the jejunum showed marked crypt elongation and reduced villus height (Figure 2B). The villi returned to normal height on day 7, lengthened on days 8 and 9, and then shortened gradually toward normality, post day 9. Crypt depth declined on day 7 and normalized by day 11 (Figure 2B). To eliminate the possibility that the above histological changes were a result of a reduced food intake following methotrexate injection, groups of pair-fed rats without methotrexate injection were killed on day 5 and day 8 for comparison. Histological analysis revealed normal intestinal villus height and crypt depth in the pair-fed rats on days 5 and 8 (not shown), indicating that the intestinal histological changes in the methotrexate-injected animals did not result from a reduction in food intake, but rather were a result of a direct effect of methotrexate on the intestinal mucosa.

At the cellular level, a significant drop of crypt cell BrdU labelling on day 3 (Figure 1 E, F and Figure 2C) preceded the histological damage (villus shortening and some crypt loss) which peaked on day 5. Conversely, an up-regulation of crypt cell proliferation on days 5 and 6 (Figures 1G and 2C) occurred 1 or 2 days prior to the elongation of crypts and villi (Figure 2B, C), suggesting that a hyperproliferation of crypt cells led to the regeneration of the intestine.

To begin to examine the potential role of HGF during intestinal regeneration following methotrexate-induced damage, mRNA and protein expression of both HGF and receptor c-met was examined in the proximal jejunal tissues throughout the methotrexate-induced damage/repair time course. By ribonuclease protection assay, expression of HGF mRNA in the intestine was found to be very low compared to that of c-met, necessitating far longer exposure of autoradiographs. This is evidenced by the much lower expression level with normal jejunal RNA (day 0) compared to that of liver RNA (used as a positive control for HGF expression) (Figure 3A). However, the level of c-met mRNA was found to be comparable in both tissues (liver data not shown). In the time course study (Figure 3A) HGF mRNA was increased in the jejunum on day 5 and day 6, a response that was statistically significant in day 5 animals compared to the day 0 controls ($P < 0.05$) (Figure 3C). From day 7, the HGF mRNA level declined to below the level of normal controls, with the decrease on day 8 being statistically significant ($P < 0.05$). By comparison, c-met mRNA was increased from day 4 to day 8 with statistical significance attained in each case (Figure 3B, 3C). Compared to HGF, up-regulation of c-met mRNA was of greater magnitude, with the peak increase in c-met observed on day 6, 175% of day 0 expression. From day 7, the c-met mRNA level began to decline, and had almost reached to the normal control level by day 9 (Figure 3C).

In situ hybridization was employed in concert to localize the changes in HGF and c-met mRNA expression within the jejunal tissue during the damage/repair time course (Figure 4). Using sense riboprobes as negative controls, very little signal was observed (Figure 4A for HGF, Figure 4E for c-met). Using antisense riboprobes, in the normal jejunum weak to moderate signal was detected for both HGF (Figure 4B) and c-met (Figure 4F), the majority of which was localized cytoplasmically to the intestinal crypt (more on the luminal side than on the basolateral side) and lower villus epithelium, with little or no signal in the villus tips, confirming a previous in situ hybridization study for HGF (Wang et al, 1994). Consistent with increases in mRNA quantitated by ribonuclease protection assays, there were increases in both HGF (Figure 4C) and c-met (Figure 4G) mRNA hybridization signals in day 5 rats after methotrexate treatment. Expression appeared to be localized evenly to all cell types throughout the crypt epithelium and at lower levels in cells of the lower villus epithelium, as per the normal intestine. On day 5 and 6, increased HGF (Figure 4D) and c-met mRNA (Figure 4H) expression could also be seen in immune cells (as judged by cell morphology) of the lamina propria. However, the specific cell-types and biological implications of this expression remain to be determined.

Western blotting analysis was performed on the total protein samples extracted from the tissue specimens used for the RNA extraction. Under reducing conditions, tissue HGF as well as the recombinant HGF standard were shown as the 70 kDa bands on the Western blots probed with an antibody raised against the 70 kDa subunit (not shown). Comparisons of protein samples from different animals throughout the time course (based on equal loading of total protein per lane) revealed an up-regulation of HGF

© 2000 Cancer Research Campaign

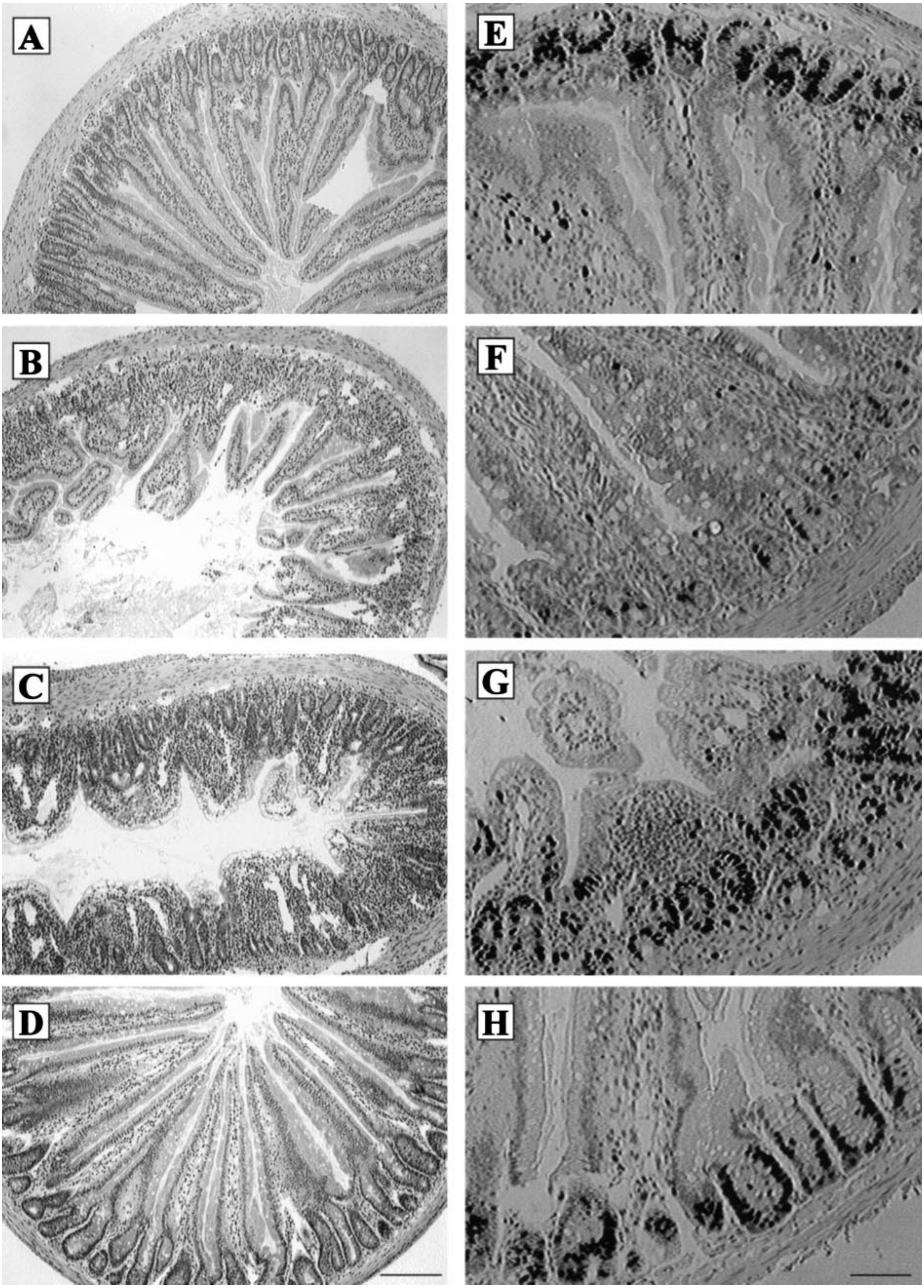

**Figure 1** Histopathological (H&E staining) and proliferative (BrdU labelling) features of proximal jejunum of rats during methotrexate-induced damage and repair. (**A** and **E**) Normal jejunal histology and BrdU labelling respectively on day 0. (**B** and **F**) A partial damage of the jejunum and a significant decrease in BrdU labelling in day 3 rats. (**C** and **G**) Severe mucosal damage and a marked up-regulation of BrdU labelling in the damaged jejunum on day 5. (**D** and **H**) Near normal histology and normal BrdU labelling on day 7. (Scale bar in **D**, 100 μm, applies to **A**–**D**; bar in **H**, 35 μm, applies to **E**–**H**.)

*© 2000 Cancer Research Campaign*

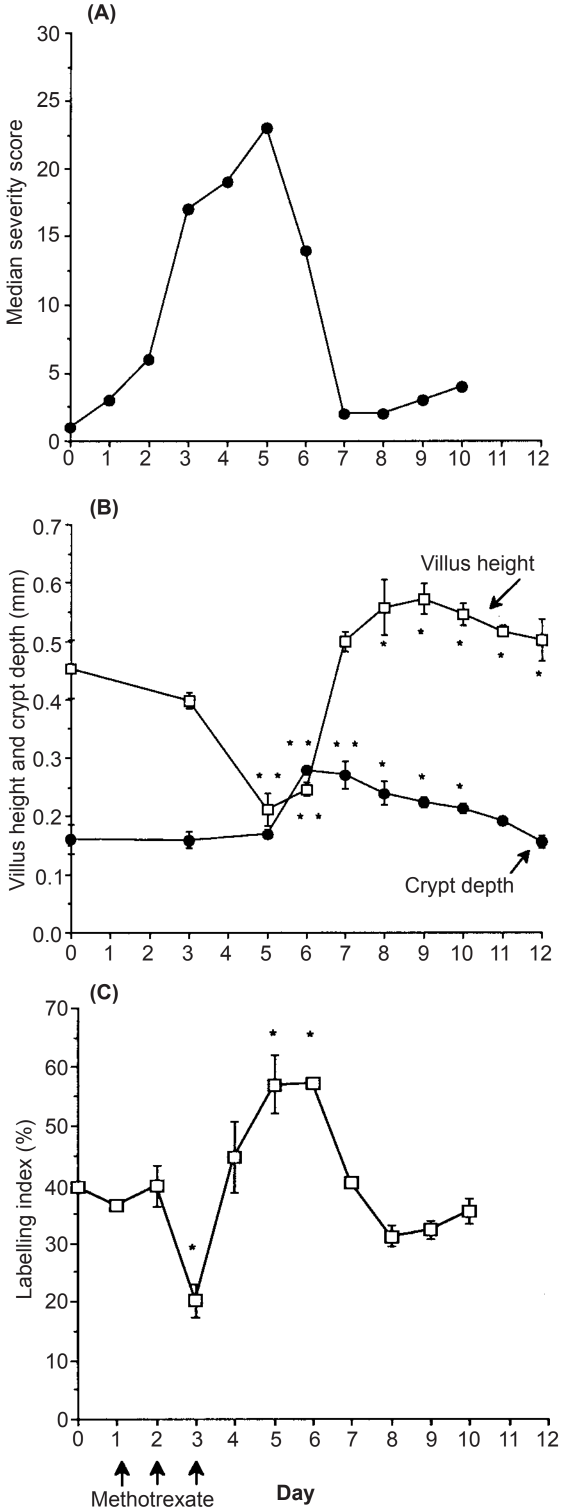

**Figure 2** Quantitative profiles of histopathological features and BrdU labelling of the proximal jejunum during methotrexate-induced damage and repair. (**A**) Semi-quantitative scoring of damage severity, showing the median severity score (*n* = 8). (**B**) Villus height and crypt depth measurements (mean ± s.e.m., *n* = 4), showing villus shortening during days 5 and 6, crypt elongation starting on day 6 and villus lengthening beginning at day 7, followed by a gradual process of normalization. The symbols ** and * respectively indicate statistical significance at $P < 0.01$ and $P < 0.05$ levels compared to day 0 controls. (**C**) The profile of BrdU labelling index of the time course (means ± s.e.m., *n* = 4), illustrating a significant (*) decrease on day 3 ($P < 0.01$) and an up-regulation of labelling on days 5 and 6 ($P < 0.01$) compared to the day 0 normal control

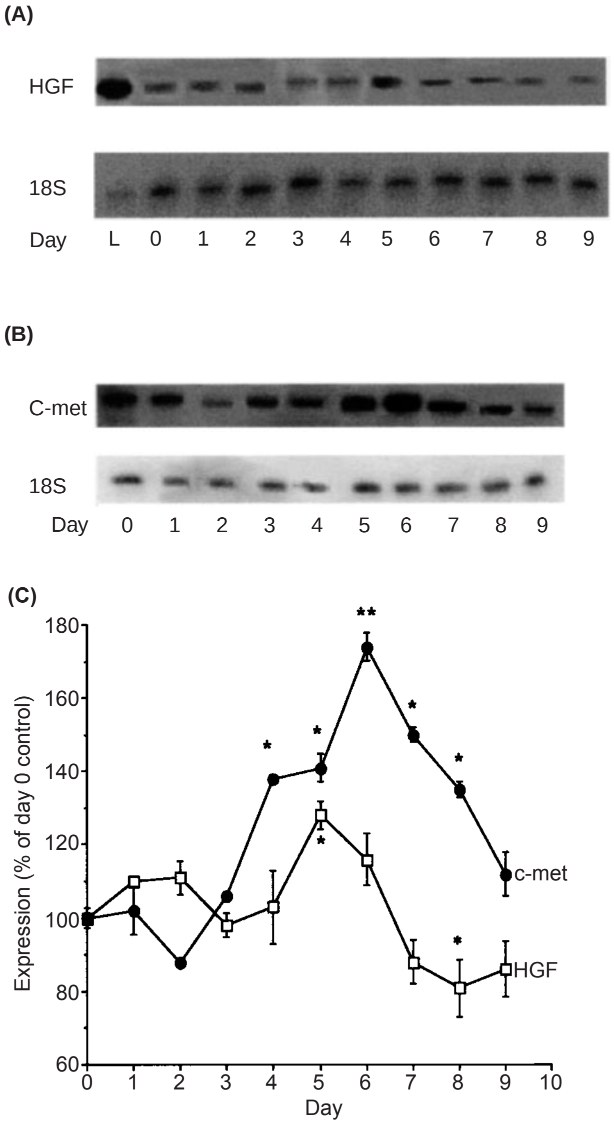

**Figure 3** Examination of HGF and c-met mRNA expression by ribonuclease protection assays in the proximal jejunum during methotrexate-induced damage and subsequent repair. Total proximal jejunal or liver RNA (L) (60 µg) was hybridized with either HGF or c-met riboprobe, and hybrids were digested with RNAase A + T1 and visualized by autoradiography after being separated on 6% PAGE gels. An 18S rRNA probe was used to control for loading. (**A** and **B**) Representative protection assays for HGF and c-met respectively. (**C**) Quantitative analysis of results following densitometry of autoradiographs. Results were normalized for loading relative to 18S rRNA and expressed as a % of day 0 levels. Asterisks depict statistical differences compared to the day 0 control (*$P < 0.05$; **$P < 0.01$)

on days 4, 5 and 6 (Figure 5A illustrating a representative blot), with the highest level (up to 225% of day 0 control) observed on day 5 as estimated by densitometry (Figure 5C). Similarly, Western blotting analyses for c-met under reducing conditions (Figure 5B) revealed that the c-met protein, shown mainly as the 145 kDa band using an antibody raised against the 145 kDa subunit (and to a lesser extent the 170 kDa band probably due to incomplete reduction of proteins), increased in level from days 3 to 6 (Figure 5C), consistent with the increase in mRNA level as measured by the ribonuclease protection assay.

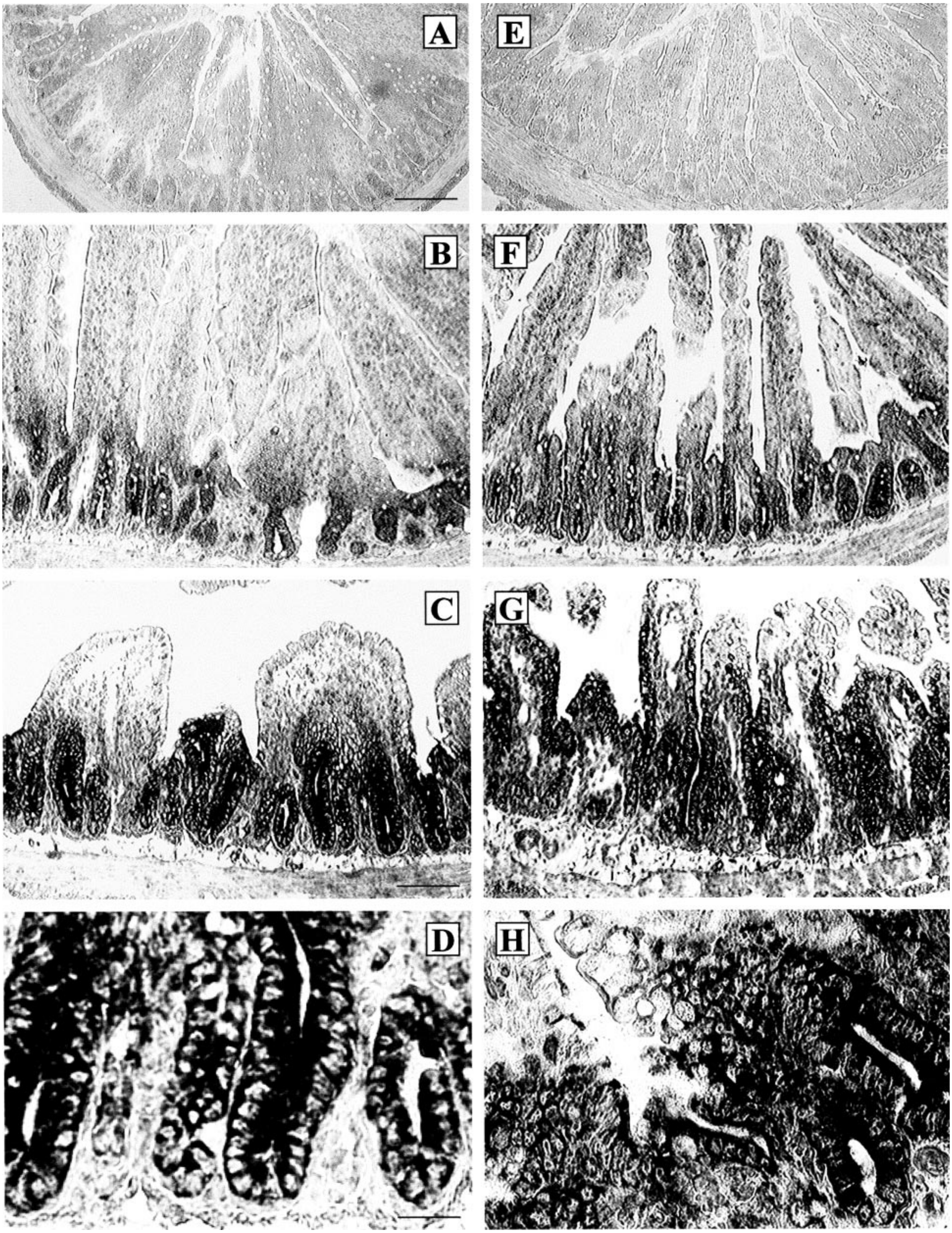

**Figure 4** In situ hybridization detection of HGF and c-met mRNA in proximal jejunum in normal and methotrexate-damaged rats. (**A** and **E**) Negative controls with HGF and c-met sense riboprobes. (**B** and **F**) Weak or moderate labelling signals (black in colour) of HGF and c-met mRNA respectively in the normal jejunum. (**C** and **G**) More intense staining for HGF and c-met mRNA respectively in methotrexate-damaged jejunum (day 5). (**D** and **H**) A higher-power view of **C** and **G** respectively. (Scale bar in **A**, 100 μm, applies to **A**, **E**; bar in **C**, 50 μm, applies to **B**, **C**, **F**, **G**; bar in **D**, 25 μm, applies to **D**, **H**)

*© 2000 Cancer Research Campaign*

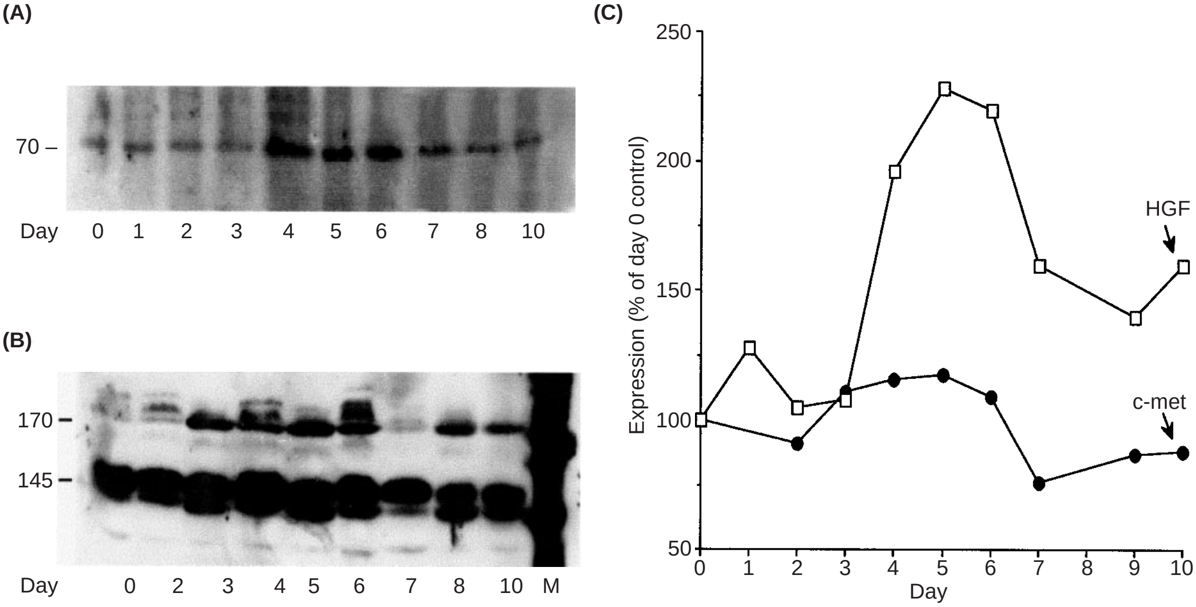

**Figure 5** Representative Western blot analyses of HGF (**A**) and c-met (**B**) in protein samples from proximal jejunum during the methotrexate-induced time course of intestinal damage and repair. For comparisons between animals of different days, equal amounts of total protein (200 μg) were loaded. Densitometry analysis of the representative blots (**C**) show the higher immunoreactivities of HGF (70 kDa) during days 4–6 and c-met (showing combined results for the 145 and 170 kDa bands) during days 3–6. (M – molecular weight markers)

### DISCUSSION

We have demonstrated that the intestinal mucositis induced by chemotherapy drug methotrexate is an acute and transient process of mucosal damage and regeneration. This process was found to be characterized by an initial significant decrease in crypt cell proliferation on day 3, preceding the histopathological features of crypt loss, villus shortening and atrophy with the severity peaking on day 5. The repair process commenced on day 4, when the crypt cell proliferation had returned to the normal level, and accelerated on days 5 and 6 as evidenced by marked up-regulation of crypt cell production. The overshoot of crypt cell proliferation on days 5 and 6 preceded the crypt elongation which commenced on day 6 followed by increases in villus height beginning on day 7. Finally, the regeneration continued after day 7 with normalization of crypt depth and villus height, with the crypt depth returning to the normal level by day 11. This study confirms the original findings on the adverse side-effects of methotrexate as a chemotherapeutic agent on intestinal epithelial renewal. Methotrexate exerts its toxicity by directly inhibiting DNA synthesis, leading to a reduction of mitosis in the crypts and shortening of the villi (Trier, 1962; Altmann, 1974), followed by proliferative recovery phase after the damage (Taminiau et al, 1980).

Previous in vivo studies have demonstrated that HGF plays an important role in gastric ulcer repair, and in vitro studies have shown that HGF stimulates intestinal cell proliferation and restitution (Kinoshita et al, 1997; Goke et al, 1998; Nishimura et al, 1998). Despite these reports, limited in vivo studies have been undertaken to investigate the role of HGF in intestinal growth and repair (Kato et al, 1998). In the present study, we have demonstrated up-regulation of both HGF and c-met during the repair of the small intestinal mucosa following methotrexate-induced damage in the rat, similar to that observed during the healing of gastric ulcers (Tsujii et al, 1994; Kinoshita et al, 1995; Schmassmann et al, 1997*b*). The transient up-regulation in HGF mRNA and protein from days 4 to 6, peaking on day 5 coincides with the timing of hyperproliferation during the repair of the intestine. Similarly, the levels of c-met protein and mRNA were elevated from days 3 or 4 to 6 or 8, peaking on day 6. The coincident timing of HGF up-regulation and crypt cell hyperproliferation strongly suggests an involvement of HGF in the stimulation of crypt cell proliferation as a prelude to the intestinal mucosal regeneration. This finding is consistent with previous in vitro studies reporting HGF to be a potent mitogen for intestinal epithelial cells (Dignass et al, 1994; Fukamachi et al, 1994). Our study is also consistent with in vivo studies demonstrating that exogenous HGF increased intestinal mass and function after massive small bowel resection (Kato et al, 1998). Furthermore, our in situ hybridization showed a co-localization of HGF and c-met mRNA in the crypt and lower villous epithelium in both the normal and regenerating intestine and an up-regulation of staining intensity during the repair phase, suggesting an autocrine or a paracrine mechanism of action for HGF in crypt cell proliferation and possibly cell migration during maintenance and repair of intestinal epithelium.

A numbers of growth factors and cytokines have been implicated in intestinal regeneration in various animal models of intestinal damage, including transforming growth factor α and β (Barnard et al, 1995; Dignass et al, 1996), insulin-like growth factor (Howarth et al, 1998), intestinal trefoil factor (Poulsom, 1996) and keratinocyte growth factor (Farrell et al, 1998). In this study, we observed a close histopathological resemblance between the methotrexate-induced rat intestinal mucositis and the clinical gut mucositis seen in chemotherapy patients, a close temporal correlation between the upregulation of HGF and its receptor and the hyperproliferative repair, and a co-localization of HGF and c-met mRNA to intestinal crypt epithelium. These results suggest that HGF may play an important role in stimulating intestinal repair after acute damage and might be therapeutically useful in enhancing intestinal repair in clinical settings. Further studies, which could include the use of specific inhibition of the action of HGF or c-met by using neutralizing antibody, antisense RNA, or HGF/c-met mutation or other approaches, need to be conducted to test whether the HGF/c-met ligand/receptor system plays an essential role in mucosal cell proliferation in vivo during intestinal repair. A recent report has shown that exogenous HGF increased intestinal mass and function after massive small bowel resection (Kato et al, 1998), supporting this potential role in intestinal repair.

### ACKNOWLEDGEMENTS

This project was funded in part by a Cooperative Research Centre grant from the Australian Government and a project grant (to CJX and LCR) from the Australian National Health and Medical Research Council (NHMRC). The authors thank C Mardell, J Cool, D Tran and C Tran for technical assistance.

### REFERENCES

Altmann GG (1974) Changes in the mucosa of the small intestine following methotrexate administration or abdominal X-irradiation. *Am J Anat* **140**: 263–280

Barnard JA, Beauchamp RD, Russell WE, Dubois RN and Coffey RJ (1995) Epidermal growth factor-related peptides and their relevance to gastrointestinal pathophysiology. *Gastroenterology* **108**: 564–580

Costa G and Donaldson SS (1979) Current concepts in cancer. Effects of cancer and cancer treatment on the nutrition of the host. *N Engl J Med* **300**: 1471–1474

Di Renzo MF, Narsimhan RP, Olivero M, Bretti S, Giordano S, Medico E, Gaglia P, Zara P and Comoglio PM (1991) Expression of the Met/HGF receptor in normal and neoplastic human tissues. *Oncogene* **1997**: 2003

Dignass AU, Lynch-Devaney K, Podolsky DK (1994) Hepatocyte growth factor/scatter factor modulates intestinal epithelial cell proliferation and migration. *Biochem Biophys Res Commun* **202**: 701–709

Dignass AU, Stow JL and Babyatsky MW (1996) Acute epithelial injury in the rat small intestine in vivo is associated with expanded expression of transforming growth factor α and β. *Gut* **38**: 687–693

Donaldson SS and Lenon RA (1979) Alterations of nutritional status: the impact of chemotherapy and radiation therapy. *Cancer* **43** (Suppl.): 2036–2053

Farrell CL, Bready JV, Rex KL, Chen JN, DiPalma CR, Whitcomb KL, Yin S, Hill DC, Wiemann B, Starnes CO, Havill AM, Lu Z-N, Aukerman SL, Pierce GF, Thomason A, Potten CS, Ulich TR and Lacey DL (1998) Keratinocyte growth factor protects mice from chemotherapy and radiation-induced gastrointestinal injury and mortality. *Cancer Res* **58**: 933–939

Fox JG, Li X, Cahill RJ, Andruits K, Rustgi AK, Odze R and Wang TC (1996) Hypertrophic gastropathy in *Helicobacter felis*-infected wild-type C57BL/6 mice and p53 homizygous transgenic mice. *Gastroenterology* **110**: 155–166

Fukamachi F, Ichinose M, Tsukada S, Kakei N, Suzuki T, Miki K, Kurokawa K and Masui T (1994) Hepatocyte growth factor specifically stimulates gastrointestinal growth in primary culture. *Biochem Biophys Res Commun* **205**: 1445–1451

Goke M, Kanai M and Podolsky DK (1998) Intestinal fibroblasts regulate intestinal epithelial cell proliferation via hepatocyte growth factor. *Am J Physiol* **37**: G809–G818

Howarth GS, Francis GL, Cool JC, Xu X, Byard RW and Read LC (1996) Milk growth factors enriched from cheese whey ameliorate intestinal damage by methotrexate when administered orally to rats. *J Nutr* **126**: 2519–2530

Howarth GS, Cool JC, Bourne AJ, Ballard FJ and Read LC (1998) Insulin-like growth factor-I (IGF-I) stimulates regrowth of the damaged intestine in rats, when administered following, but not concurrent with, methotrexate. *Growth Factors* **15**: 279–292

Jolivet J, Wowan JH, Curt GA, Clendennin NJ and Charner BA (1983) The pharmacology and clinical use of methotrexate. *N Engl J Med* **309**: 1094–1104

Kato Y, Yu D and Schwartz MZ (1998) Enhancement of intestinal adaptation by hepatocyte growth factor. *J Pediatr Surg* **33**: 235–239

Kermorgant S, Walker F, Hormi K, Dessirier V, Lewin MJ and Lehy T (1997) Developmental expression and functionality of hepatocyte growth factor and c-Met in human fetal digestive tissues. *Gastroenterology* **112**: 1635–1647

Kinoshita Y, Nakata H, Hassan S, Asahara M, Kawanami C, Matsushima Y, Naribayashi-Inomoto Y, Ping CY, Min D, Nakamura A and Chiba T (1995) Gene expression of keratinocyte and hepatocyte growth factors during the healing of rat gastric mucosal lesions. *Gastroenterology* **109**: 1068–1077

Kinoshita Y, Kishi K, Asahara M, Matsushima Y, Wang H-Y, Miyazawa K, Kitamura N and Chiba T (1997) Production and activation of hepatocyte growth factor during the healing of rat gastric ulcers. *Digestion* **58**: 225–231

Laemmli UK (1970) Cleavage of the structural proteins during the assembly of the head of the bacteriophage T4. *Nature* **227**: 680–685

Liu YH, Tolbert EM, Sun AM and Dworkin LD (1996) Primary structure of rat HGF receptor and induced expression in glomerular mesangial cells. *Am J Physiol* **40**: F679–F688

Nishimura S, Takahashi M, Ota S, Hirano M and Hiraishi H (1998) Hepatocyte growth factor accelerates restitution of intestinal epithelial cells. *J Gastroenterol* **33**: 172–178

Poulsom R (1996) Trefoil peptides. *Baillière's Clin Gastroenterol* **10**: 113–134

Prat M, Narshiman RP, Crepaldi T, Nicotra MR, Natali PG and Comoglio PM (1991) The receptor encoded by the human c-met oncogene is expressed in hepatocytes, epithelial cells and solid tumors. *Int J Cancer* **49**: 323–328

Schmassmann A, Hirschi C, Stettler C, Poulsom R and Halter F (1997*a*) Recent work with hepatocyte growth/scatter factor. In: *The Gut as a Model in Cell and Molecular Biology*, Halter F, Winton D and Wright NA (eds), pp. 180–193. Kluwer Academic Publishers: Lancaster

Schmassmann A, Stettler C, Poulsom R, Tarasova N, Hirschi C, Flogerzi B, Matsumoto K, Nakamura T and Halter F (1997*b*) Roles of hepatocyte growth factor and its receptor met during gastric ulcer healing in rats. *Gastroenterology* **113**: 1858–1872

Takahashi M, Ota S, Hata Y, Ogura K, Kurita M, Terano A, Nakamura T and Omata M (1996) Constitutive expression of hepatocyte growth factor may maintain the sheet construction of gastric epithelial cells through facilitating actin-myosin contractile system. *Biochem Biophys Res Commun* **214**: 40–46

Taminiau JA, Gall DG and Hamilton JR (1980) Response of the rat small intestine epithelium to methotrexate. *Gut* **21**: 486–492

Trier JS (1962) Morphologic alterations induced by methotrexate in the mucosa of human proximal intestine. *Gastroenterology* **42**: 295–305

Tsujii M, Kawano S, Tsuji S, Ito T, Hayashi N, Horimoto M, Mita E, Nagano K, Masuda E, Fusamoto H and Kamada T (1994) Increased expression of c-met messenger RNA following acute gastric injury in rats. *Biochem Biophys Res Commun* **200**: 536–541

Wang Y, Selden C, Farnaud S, Calnan D and Hodgson HJF (1994) Hepatocyte growth factor (HGF/SF) is expressed in human epithelial cells during embryonic development; studies by in situ hybridisation and Northern blot analysis. *J Anat* **185**: 543–551

Wolf HK, Zarnegar R and Michalopoulos GK (1991) Localization of hepatocyte growth factor in human and rat tissues: an immunohistochemical study. *Hepatology* **14**: 488–494

Zhou X-F, Deng YS, Chie ET, Xue Q, Zhong J-H, McLachlan EM, Rush RA and Xian CJ (1999) Satellite cell-derived nerve growth factor or neurotrophin-3 are involved in noradrenergic sprouting in the dorsal root ganglia following peripheral nerve injury in the rat. *Eur J Neurosci* **11**: 1711–1722

 © 2000 Cancer Research Campaign
